# Linear Discrete Pursuit Game with Phase Constraints

**DOI:** 10.1155/2014/435103

**Published:** 2014-10-30

**Authors:** Asqar Raxmanov, Gafurjan Ibragimov

**Affiliations:** ^1^Department of Informatics, Tashkent University of Information Technologies, (TUIT), Amir Temur Street 108, 100202 Tashkent, Uzbekistan; ^2^Institute for Mathematical Research & Department of Mathematics, FS, Universiti Putra Malaysia, 43400 Serdang, Selangor, Malaysia

## Abstract

We consider a linear pursuit game of one pursuer and one evader whose motions are described by different-type linear discrete systems. Position of the evader satisfies phase constraints: *y* ∈ *G*, where *G* is a subset of *R* ^*n*^. We considered two cases: (1) controls of the players satisfy geometric constraints, and (2) controls of the players satisfy total constraints. Terminal set *M* is a subset of *R* ^*n*^ and it is assumed to have a nonempty interior. Game is said to be completed if *y*(*k*) − *x*(*k*) ∈ *M* at some step *k*; thus, the evader has not the right to leave set *G*. To construct the control of the pursuer, at each step *i*, we use the value of the control parameter of the evader at the step *i*. We obtain sufficient conditions of completion of pursuit from certain initial positions of the players in finite time interval and construct a control for the pursuer in explicit form.

## 1. Introduction

A number of works were devoted to investigate differential and discrete pursuit games with various constraints on controls (see, e.g., [1–8]). Differential games with phase constraints on a position of one or several players are studied in many works (see, e.g., [9–18]). Most of these works were devoted to studying simple motion pursuit-evasion game problem. For example, in [10], an evasion problem is solved, when the evader moves on a curve and the pursuer moves on the plane. In this work, it is assumed that the maximum speed of the pursuer is equal to 1, and the maximum speed of the evader is more than 1. In the work [11], a simple motion pursuit-evasion game problem of many pursuers and one evader is considered on a compact set. The main result of this paper is estimation for guaranteed pursuit time. The work [12] is devoted to studying a game problem on a convex closed set when controls of players satisfy total constraints. In [13], simple motion pursuit game problem is solved for all initial positions of space when the maximum speeds of players are equal and the evader moves on a convex bounded set with nonempty interior. In [15], sufficient conditions of completion of pursuit are obtained for a linear differential game when the evader moves in a bounded convex set.

In [16], the case, where the terminal and control sets are convex compact sets, is studied. In [17], necessary and sufficient conditions of solvability of the evasion problem in a convex polyhedron were received.

In the present paper, we consider a linear discrete pursuit game of one pursuer and one evader. We will study both total and geometric constraints on controls of players under assumption that the terminal set *M* consists of an interior point in *R* ^*n*^. The evader can move only in a given bounded convex set. Some sufficient conditions of completion of pursuit have been obtained.

## 2. Statement of the Problem

Consider a discrete game described by the equations

(1) x(k)=Ax(k)+U(k),

(2) y(k)=By(k−1)+V(k),

where *x*, *y*, *u*, *v* ∈ *R* ^*n*^, *n* ≥ 1, *k* = 1,2,…, *k* is step number, *A*, *B* are constant square matrices of order *n*, and *U* and *V* are control parameters of the pursuer and evader, respectively. Control parameters *U* and *V* are constructed in the form of sequences *U* = *U*(·) = {*U*(1), *U*(2),…, *U*(*k*),…} and *V* = *V*(·) = {*V*(1), *V*(2),…, *V*(*k*),…} which satisfy the following geometric constraints:

(3) $$\begin{array}{c} \left|U\left(k\right)\right|\leq \rho ,\;\rho >0, \end{array}$$

(4) $$\begin{array}{c} \left|V\left(k\right)\right|\leq \sigma ,\;\sigma >0, \end{array}$$

or the total constraints

(5) $$\begin{array}{c} \overset{\infty }{\underset{k=1}{\sum }}{\left|U\left(k\right)\right|}^{2}<{\rho_{1}}^{2}, \end{array}$$

(6) $$\begin{array}{c} \overset{\infty }{\underset{k=1}{\sum }}{\left|V\left(k\right)\right|}^{2}<{\sigma_{1}}^{2}. \end{array}$$

The pursuer moves according to (1) with control parameter *U*, and the evader moves according to (2) with control parameter *V*. The purpose of the pursuer is inclusion realization:

(7) $$\begin{array}{c} y\left(k\right)-x\left(k\right)\in M, \end{array}$$

for some final step *k*, and the evader's purpose is opposite. State of the evader is subjected to constraint *y* ∈ *G*, where *G* is a bounded convex set in *R* ^*n*^. It is assumed that the terminal set *M* is a subset of *R* ^*n*^ and it has nonempty interior. The condition int⁡*M* ≠ *Ø* implies that there are a number *l* > 0 and vector *m* ∈ *M* to satisfy the inclusion *lS* ⊂ −*m* + *M*, where *S* is the ball of radius 1 centered at the origin. Since *G* is a bounded convex set, there exists a number *R* > 0 such that *G* ⊂ *RS*.

**Definition 1 .**

Admissible control of the pursuer (evader) is defined as a sequence *U* = *U*(·)(resp.  *V* = *V*(·)) that satisfies the constraint (3) (or (5)) (resp., (4) (or (6))).

**Definition 2 .**

A sequence

(8) U=(U(1),U(2),…),U(k)=U(x(k),y(k),V(k)),U(k):R3n⟶Rn,

where *V* = (*V*(1), *V*(2),…, *V*(*k*),…) is an admissible control of the evader, is called strategy of the pursuer in the games (1)–(4) or (1)-(2), (5)-(6).

**Definition 3 .**

If for an initial position *z*_0_ = (*x*_0_, *y*_0_), *y*_0_ − *x*_0_ ∉ *M*, a strategy of the pursuer, and any admissible control of the evader the solutions of (1), (2)

(9) $$\begin{array}{c} x=x\left(\cdot \right)=\left\{x\left(1\right),x\left(2\right),\ldots ,x\left(k\right),\ldots \right\},\\ y=y\left(\cdot \right)=\left\{y\left(1\right),y\left(2\right),\ldots ,y\left(k\right),\ldots \right\} \end{array}$$

satisfy the inclusion (7) at some *k* ≤ *N* = *N*(*z*_0_), then we say that pursuit can be completed for *N* steps.

**Problem 4 .**

Find sufficient conditions of completion of pursuit in the games (1)–(4).

**Problem 5 .**

Find sufficient conditions of completion of pursuit in the games (1)-(2), (5)-(6).

## 3. Main Result

### 3.1. The Case of Geometric Constraints

**Assumption 6 .**

Let the following conditions be satisfied:

1. *B* = *A*,
2. *ρ* > *σ*/*α*, where *α* = *R*/(*R* − *l*).

**Theorem 7 .**

Let Assumption 6 hold and, for the position *z*_0_, at some step *k* = *N*(*z*_0_), the inclusion

(10) −m+Ak(1αy0−x0) ∈(ρ−σα)(Ak−1+Ak−2+⋯ +A+E)S

holds true. Then pursuit can be completed in the games (1)–(4) from initial position *z*_0_ for *N*(*z*_0_) steps.

**Proof**

Let *V* = *V*(*k*),  *k* = 1,2,…, be an arbitrary admissible control of the evader. Set

(11) $$\begin{array}{c} U\left(k\right)=\frac{1}{\alpha }V\left(k\right)+w, \end{array}$$

where the vector *w* ∈ (*ρ* − (*σ*/*α*))*S* will be specified later. Then for the solutions of (1), (2) with initial conditions *x*(0) = *x*_0_, *y*(0) = *y*_0_, we have

(12) y(k)−x(k)=Aky0−Akx0 −(Ak−1+Ak−2+⋯+A+E)w +(1−1α)(Ak−1V(1)+Ak−2V(2)      +⋯+AV(k−1)+V(k))=Ak(1αy0−x0) −(Ak−1+Ak−2+⋯+A+E)w +(1−1α)(Aky0+Ak−1V(1)+Ak−2V(2)      +⋯+AV(k−1)+V(k)).

Consider the equation

(13) −m+AN(1αy0−x0) =(AN−1+AN−2+⋯+A+E)w

with respect to the unknown vector *w* ∈ (*ρ* − *σ*/*α*)*S*. It follows from the inclusion (10) that there exists a solution of (13). Denote it by $\tilde{w}$. In (12), letting $w=\tilde{w}$ and using *y* ∈ *RS* we obtain at *k* = *N* that

(14) |−m+y(N)−x(N)| ≤|(1−1α)(ANy0+AN−1V(1)+AN−2V(2)      +⋯+AV(N−1)+AV(N))|  +|−m+AN(1αy0−x0)   −(AN−1+AN−2+⋯+A+E)w~| =|(1−1α)y(N)| =(1−R−lR)|y(N)|≤lRR=l.

Therefore, *y*(*N*) − *x*(*N*) ∈ *M*.

For the pursuit control *U*(*k*), we have

(15) |U(k)|=|1αV(k)+w~|≤1α|V(k)|+|w~|≤σα+(ρ−σα)=ρ.

The proof is complete.

**Assumption 8 .**

Let the following conditions be satisfied:

1. det⁡(*A*) ≠ 0 and there exists a number *d* > 0 such that ||(*A* ^*k*^)^−1^ *B* ^*k*^|| ≤ *d* for any *k* ≥ 1, where *A* ^−1^ is the inverse of the matrix *A* and ||*A*|| is operator norm of the matrix *A*;
2. *ρ* > *dσ*/*α*.

**Theorem 9 .**

Let Assumption 8 hold and for the position *z*_0_ there exists a step *N*(*z*_0_) such that at *k* = *N*(*z*_0_) the inclusion

(16) −m+1αBky0−Akx0 ∈(ρ−dσα)(Ak−1+Ak−2+⋯+A+E)S

holds true. Then pursuit can be completed in the games (1)–(4) from the initial position *z*_0_ for *N*(*z*_0_) steps.

**Proof**

Let the pursuer use the following strategy:

(17) $$\begin{array}{c} U\left(i\right)=\frac{1}{\alpha }{\left(A^{k-i}\right)}^{-1}B^{k-i}V\left(i\right)+w, \end{array}$$

where *V* = *V*(*i*), *i* = 1,2,…, *k*, *k* > 1, is any admissible control of the evader, and *w* ∈ (*ρ* − *dσ*/*α*)*S* is specified later. Then for the solutions of (1), (2) with initial conditions *x*(0) = *x*_0_, *y*(0) = *y*_0_ we have

(18) −m+y(k)−x(k) =−m+Bky0+Bk−1V(1)+Bk−2V(2)  +⋯+BV(k−1)+V(k)−Akx0  −1α[Ak−1(Ak−1)−1Bk−1V(1)    +Ak−2(Ak−2)−1Bk−2V(2)    +AA−1BV(k−1)+V(k)]  −(Ak−1+Ak−2+⋯+A+E)w =−m+1αBky0−Akx0  −(Ak−1+Ak−2+⋯+A+E)w  +(1−1α)y(k).

Consider the equation

(19) −m+1αBNy0−ANx0 =(AN−1+AN−2+⋯+A+E)w

with respect to the unknown vector *w* ∈ (*ρ* − *dσ*/*α*)*S*. It follows from the inclusion (16) of Theorem 9 that there exists a solution of (19). Let us denote it by $\overset{˘}{w}$. Since *y* ∈ *G* ⊂ *RS*, letting $w=\overset{˘}{w}$ in (18), we obtain

(20) |−m+y(N)−x(N)| =(1−1α)|y(N)|≤(1−1α)R≤l.

Hence, pursuit can be completed in the games (1), (2). Next, for *U*(*i*), *i* ≥ 1, using the conditions (1), (2) of Assumption 8, we can see from (17) that

(21) |U(i)|≤1α|A(N−i)−1BN−iV(i)| +|w^|≤1α||A(N−i)−1BN−i|||V(i)| +|w^|≤1αdσ+ρ−dσα=ρ.

Proof of Theorem 9 is complete.

### 3.2. The Case of Total Constraints on Controls

Let us consider the games (1), (2) with total constraints (5)-(6) on controls.

**Assumption 10 .**

Let the following conditions be satisfied:

1. *B* = *A*,
2. *ρ*_1_ > *σ*_1_/*α*.

**Theorem 11 .**

Let Assumption 10 hold, and for the position *z*_0_ let the inclusion

(22) −m+Ak(1αy0−x0) ∈1k(ρ1−σ1α)(Ak−1+Ak−2+⋯+A+E)S

be satisfied at some step *k* = *N*(*z*_0_). Then pursuit can be completed in the games (1), (2), (5), and (6) from the initial position *z*_0_ for *N*(*z*_0_) steps.

**Proof**

Let *V* = *V*(*k*), *k* = 1,2,…, be any admissible control of the evader. Construct the strategy for the pursuer in the following form:

(23) $$\begin{array}{c} U=U\left(k\right)=\frac{1}{\alpha }V\left(k\right)+w, \end{array}$$

where *w* is a vector in $\left(1/\sqrt{N}\right)\left(\rho_{1}-\sigma_{1}/\alpha \right)S$ which will be specified later. For the solutions *x*(*k*), *y*(*k*), we have in view of (1), (2) that

(24) y(k)−x(k)=Ak(y0−x0) −(Ak−1+Ak−2+⋯+A+E)w +(1−1α)(Ak−1V(1)+Ak−2V(2)      +⋯+AV(k−1)+V(k))=Ak(1αy0−x0) +(Ak−1+Ak−2+⋯+A+E)w +(1−1α)(Aky0+Ak−1V(1)+Ak−2V(2)      +⋯+AV(k−1)+V(k)).

Consider the equation

(25) $$\begin{array}{c} -m+A^{N}\left(\frac{1}{\alpha }y_{0}-x_{0}\right)=\left(A^{N-1}+A^{N-2}+\cdots +A+E\right)w \end{array}$$

with respect to the unknown vector $w\in \left(1/\sqrt{N}\right)\left(\rho_{1}-\sigma_{1}/\alpha \right)S$. According to (22), (25) has a solution. Denote by $\hat{w}$ lexicographically minimum solution of (25). In (24), letting $w=\hat{w}$ and using the condition *y* ∈ *RS*, we have at *k* = *N*

(26) |−m+y(N)−x(N)| =|(1−1α)y(N)−m+AN(1αy0−x0)   −(AN−1+AN−2+⋯+A+E)w^| =|(1−1α)y(N)| =(1−R−lR)|y(N)|≤l,

and therefore *y*(*N*) − *x*(*N*) ∈ *M*.

Next, we prove that if the evader's control *V* = *V*(*k*), *k* = 1,2,…, *N*, satisfies the constraint (6); then the pursuer's control $U(k)=\left(1/\alpha \right)V(k)+\hat{w}$, *k* = 1,2,…, *N*, satisfies the constraint (5). Indeed, by the inclusion $\hat{w}\in \left(1/\sqrt{N}\right)\left(\rho_{1}-\sigma_{1}/\alpha \right)S$ and the Minkowski inequality we have

(27) ∑k=1N|U(k)|2=∑k=1N|V(k)+w^⌢|2≤[(1α2∑k=1N|V(k)|2)1/2+(∑k=1N|w^⌢|2)1/2]2≤[σ1α+1N(ρ1−σ1α)N]2=ρ12.

The proof is complete.

**Theorem 12 .**

Let conditions (1) and (2) of Assumption 8 be satisfied at *ρ* = *ρ*_1_, *σ* = *σ*_1_ and let for point *z*_0_

(28) 1α1Bky0−Akx0 ∈1k(ρ1−dσ1α1)(Ak−1+Ak−2+⋯+A+E)S.

at some *k* = *N*(*z*_0_). Then pursuit can be completed in the games (1), (2), (5), and (6) from initial position *z*_0_ for *N*(*z*_0_) steps.

Theorem 12 can be proved as Theorems 9 and 11 in a similar fashion.

### 3.3. Example

We consider a discrete game described by the equations

(29) $$\begin{array}{c} x_{k}=x_{k-1}+u_{k},\\ y_{k}=y_{k-1}+v_{k}, \end{array}$$

where *x*_*k*_, *y*_*k*_, *u*_*k*_, *v*_*k*_ ∈ *R* ^*n*^, *n* ≥ 1, and *u* = (*u*_1_, *u*_2_,…, *u*_*k*_,…) and *v* = (*v*_1_, *v*_2_,…, *v*_*k*_,…) are controls of the pursier and evader which satisfy the constraints (3) and (4), respectively. Position of the evader is subjected to constraint *y* ∈ *rS*, *r* > 0; the terminal set is *M* = {*x*, *y*∣*x*, *y* ∈ *R* ^*n*^, *y* − *x* ∈ *lS*, *l* > 0}. For this game, *B* = *A* = *E*. Applying Theorems 7 and 9 to the game (29) yields the following result.

**Statement 1 1.**

If

(30) $$\begin{array}{c} \rho >\frac{r-l}{r}\sigma , \end{array}$$

then pursuit can be completed in the games (29), (3), and (4) from all the initial positions *x*_0_, *y*_0_ ∈ *R* ^*n*^, |*y*_0_| ≤ *r*, |*y*_0_ − *x*_0_| > *l* for finite number of steps.

Let us consider the games (29), (5), and (6). It is not difficult to verify that Theorems 11 and 12 give the following result.

**Statement 2 2.**

If

(31) $$\begin{array}{c} \rho_{1}>\frac{r-l}{r}\sigma_{1}, \end{array}$$

then pursuit can be completed in the games (29), (5), and (6) from all the initial positions *x*_0_, *y*_0_ ∈ *R* ^*n*^, |*y*_0_| ≤ *r*, |*y*_0_ − *x*_0_| > *l* for finite number of steps.

As we can see from inequalities (30) and (31) that Theorems 7 and 9 (resp., Theorems 11 and 12 in case of total constraints on controls) are applicable to the game (29) for some values of *ρ* and *σ* (resp., *ρ*_1_ and *σ*_1_) that satisfy the inequality *ρ* < *σ* (resp., *ρ*_1_ < *σ*_1_).

## 4. Conclusion

We have obtained sufficient conditions of completion of pursuit in linear discrete games, when phase constraints are imposed on position of evader. We have applied the obtained results to concrete discrete pursuit game problems with phase constraint on a position of the evader. We have constructed an example, for which pursuit problem is solvable even if speed (resource, in case of total constraints) of the pursier is a little bit less than the speed (resource) of the evader.
